# Genetic diversity of *Brucella ovis* isolates from Rio Grande do Sul, Brazil, by MLVA16

**DOI:** 10.1186/1756-0500-7-447

**Published:** 2014-07-12

**Authors:** Elaine MS Dorneles, Guilherme N Freire, Maurício G Dasso, Fernando P Poester, Andrey P Lage

**Affiliations:** 1 Departamento de Medicina Veterinária Preventiva, Escola de Veterinária, Universidade Federal de Minas Gerais, Belo Horizonte, Minas Gerais, Brazil; 2 Instituto de Pesquisas Veterinárias Desidério Finamor, Eldorado do Sul, Rio Grande do Sul, Brazil

**Keywords:** Genotyping, *Brucella ovis*, MLVA16, Ovine brucellosis

## Abstract

**Background:**

Ovine epididymitis is predominantly associated with *Brucella ovis* infection. Molecular characterization of *Brucella* spp. achieved by multi-*locus* variable number of tandem repeats (VNTR) analyses (MLVA) have proved to be a powerful tool for epidemiological trace-back studies. Thus, the aim of this study was to evaluate the genetic diversity of *Brucella ovis* isolates from Rio Grande do Sul State, Brazil, by MLVA16.

**Findings:**

MLVA16 genotyping identified thirteen distinct genotypes and a Hunter-Gaston diversity index of 0.989 among the fourteen *B. ovis* genotyped strains. All *B. ovis* MLVA16 genotypes observed in the present study represented non-previously described profiles. Analyses of the eight conserved *loci* included in panel 1 (MLVA8) showed three different genotypes, two new and one already described for *B. ovis* isolates. Among ten *B. ovis* isolates from same herd only two strains had identical pattern, whereas the four isolates with no epidemiologic information exhibited a single MLVA16 pattern each. Analysis of minimal spanning tree, constructed using the fourteen *B. ovis* strains typed in this study together with all nineteen *B. ovis* MLVA16 genotypes available in the MLVAbank 2014, revealed the existence of two clearly distinct major clonal complexes.

**Conclusions:**

In conclusion, the results of the present study showed a high genetic diversity among *B. ovis* field isolates from Rio Grande do Sul State, Brazil, by MLVA16.

## Findings

### Background

*Brucella ovis* is a rough, Gram-negative, non-spore-forming, non-motile and facultative intracellular bacterium [1]. In rams, the microorganism causes mainly epididymitis [2,3], whereas in ewes the lesions are characterized by degeneration and inflammation of the endometrium with focal or diffuse lymphoid infiltrations [4].

Infection has been recognized in all countries where sheep are of economic importance and leads to significant losses to animal production [5,6]. In Brazil, the ovine epididymitis is chiefly described in southern States (Rio Grande do Sul, Santa Catarina, Paraná), where the sheep-raising is more developed [7], having been first reported in 1966 in Rio Grande do Sul State [8]. In 1996, a clinical and serological survey of rams in Rio Grande do Sul State showed prevalence of 13.4% [9]. More recent data, with a broader sampling, (2011/2012) indicates a decrease in this prevalence index to 2.8% of positive animals [10].

Molecular characterization of *Brucella* spp. achieved by multi-*locus* variable number of tandem repeats (VNTR) analyses (MLVA) have proved to be a powerful tool to determine relationships among *Brucella* spp isolates from different animal species and from humans, as well as for epidemiological trace-back studies [11-17]. However, data regarding *B. ovis* genotyping, using MLVA16 or even other techniques are very scarce. Thus, the aim of this study was to evaluate the genetic diversity of *B. ovis* field isolates from Rio Grande do Sul, Brazil, using MLVA16.

## Methods

Fourteen *B. ovis* field isolates obtained from sheep between 1982 and 1995 were used in this study. They were provided from the collection of Instituto de Pesquisas Veterinárias Desidério Finamor and were isolated (by FPP and MGD) from semen samples collected by electroejaculation from rams in Rio Grande do Sul, Brazil (Santana do Livramento - 10; Uruguaiana - 2; and undefined municipalities - 2). All isolates from Santana do Livramento were from animals of the same herd, whereas the others four *B. ovis* isolates had not information about herd of origin. All isolates were confirmed to be *B. ovis* by biochemical and molecular tests [18-20]. Approval to use the *B. ovis* isolates in this study was formally given by the director of IPVDF.

*Brucella ovis* colonies were inactivated at 85°C for 2 hours and subjected to genomic DNA extraction [21,22]. DNA from each strain was genotyped by MLVA16, which was divided in: panel 1 (Bruce06, Bruce08, Bruce11, Bruce12, Bruce42, Bruce43, Bruce45, Bruce55); panel 2A (Bruce18, Bruce19, Bruce21); and panel 2B (Bruce04, Bruce07, Bruce09, Bruce16, Bruce30) [11,15].

From digitalized image of each gel, the band size was estimated and then converted into number of repeat units for each *locus* by using the software BioNumerics 6.1 (Applied Maths, Belgium) [15]. *Brucella melitensis* 16M (ATCC 23456^T^) was used as control for band size estimation of all MLVA16 *loci*. The genotypes obtained were compared to those deposited in the MLVAbank 2014 (http://mlva.u-psud.fr/brucella/). Clustering analysis was performed using the category coefficient and UPGMA (BioNumerics 6.1) [15]. The Hunter-Gaston diversity index (HGDI) was used [23]. The minimum-spanning tree (MST) was generated using Prim’s algorithm associated with priority rule (eBURST algorithm) and bootstrap resampling [24,25] (BioNumerics 6.1). The MST presented is the top score tree, the tree with the highest overall reliability score.

## Results

**Figure 1:**
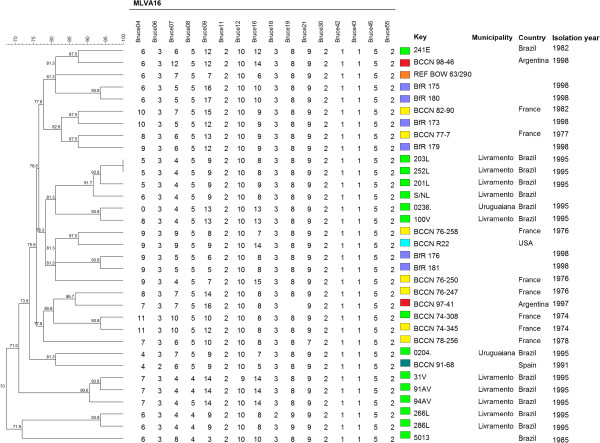
**Cluster analysis by MLVA16 genotyping of 14 *Brucella ovis *isolates from sheep of Rio Grande do Sul State, Brazil, 1982 – 1995 plus all 19 MLVA16 genotypes of *B. ovis *available in the MLVAbank 2014.** The cluster analysis was performed using the category coefficient and UPGMA (BioNumerics 6.1). Information on the origin of the isolates was color labeled.

**Figure 2:**
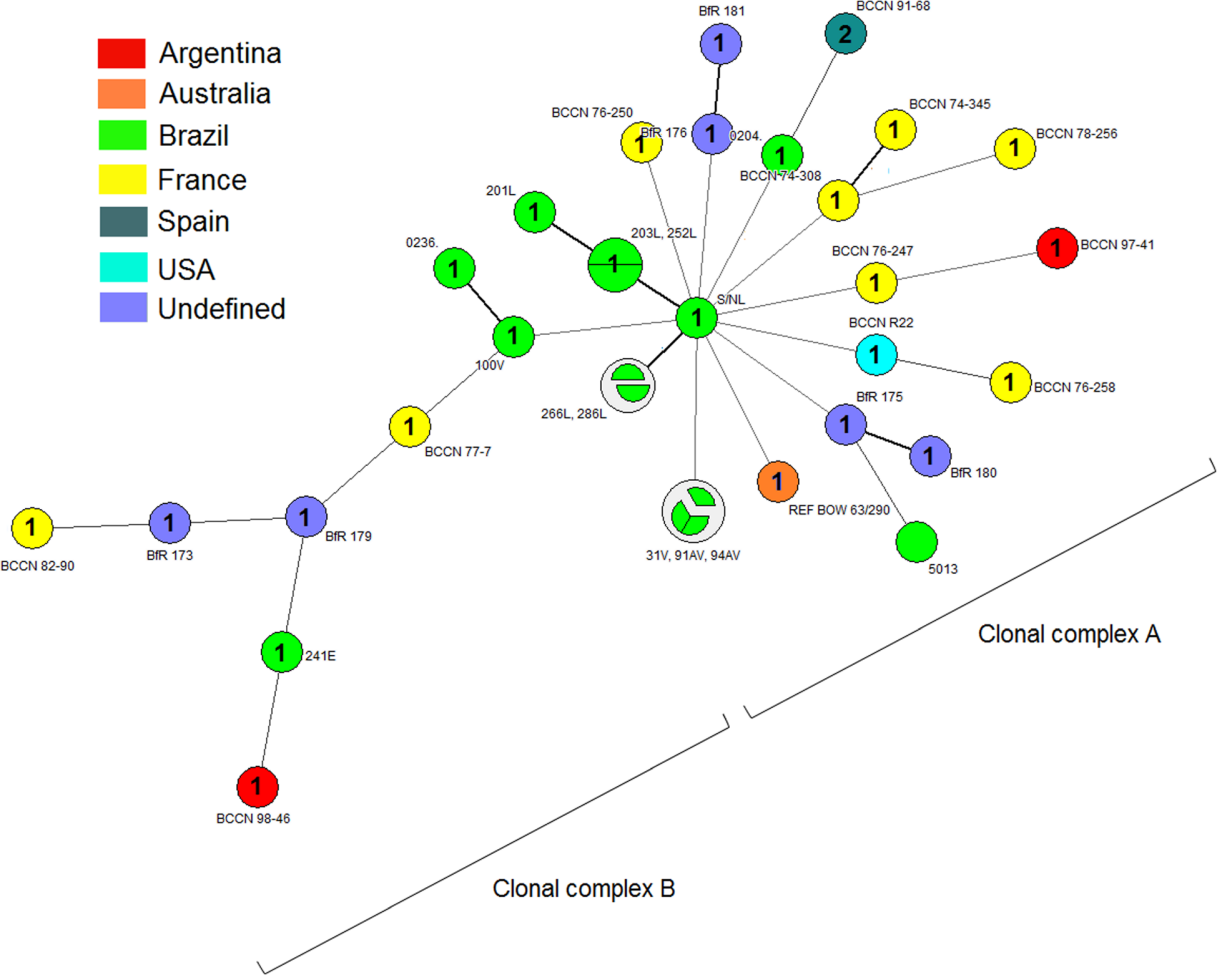
**Minimum Spanning Tree (MST) analysis of *Brucella ovis *isolates from sheep of Rio Grande do Sul State, Brazil plus all 19 MLVA16 genotypes of *B. ovis *available in the MLVAbank 2014, using the MLVA16 data.** The MST analyses included 14 *B. ovis* field strains tested in the present study (203 L, 252 L, 201 L, S/NL, 266 L, 286 L, 0236., 100 V, 0204., 241E, 31 V, 91AV, 94AV and 5013) and all nineteen *B. ovis* MLVA16 genotypes available in MLVAbank 2014 (http://mlva.u-psud.fr). The minimum spanning tree presented is the one with the highest overall reliability score and was calculated using Prim’s algorithm associated with the priority rule and the bootstrap resampling. Numbers inside each clonal complex represent the genotype on Panel 1 (MLVA8). Branch length and thickness reflects number of differences between nodes. Information on the origin of the isolates was color labeled in the same way as shown in the Figure 1.

Analysis of the MLVA16 *loci* revealed thirteen distinct genotypes among the fourteen *B. ovis* strains evaluated (Figure 1) and a HGDI of 0.989. All these MLVA16 patterns represented new genotypes, since no correspondence with those deposited on MLVAbank 2014 was found. However, the comparison of results observed in the eight conserved *loci* included in the panel 1 (MLVA8) with those available in the MLVAbank 2014 (http://mlva.u-psud.fr/brucella/) revealed that nine among the fourteen isolates had MLVA8 profile identical to profile 1 (Bruce06: 3; Bruce08: 5; Bruce11: 2; Bruce12: 10; Bruce42: 1; Bruce43: 1; Bruce45: 5; Bruce55: 2). The other five *B. ovis* isolates exhibited two different MLVA8 patterns, which were different of the MLVA8 1 and 2 genotypes (genotype 2 = Bruce06: 2; Bruce08: 5; Bruce11: 2; Bruce12: 10; Bruce42: 1; Bruce43: 1; Bruce45: 5; Bruce55: 2) (the only ones already described for *B. ovis*) due to polymorphisms in *loci* Bruce06, 08 and 12. The MST created based on MLVA16 genotypes is shown in Figure 2. Besides the *B. ovis* strains tested in the present study, all nineteen MLVA16 genotypes of *B. ovis* available in the MLVAbank 2014 were included in clustering and MST analyses. Analysis of geographical origin in the MST showed that *B. ovis* strain BCCN 98–46 from Argentina was closely related to a Brazilian *B. ovis* isolate, strain 241E (Figures 1 and 2). Moreover, MST analysis also revealed the existence of two clearly distinct major clonal complexes (clonal complexes A and B).

## Discussion

Genotyping of microorganism of great veterinary importance, such as *B. ovis,* is a valuable tool for the control of disease, since it allows the characterization of outbreaks and, the determination of the source of infection and transmission routes [26]. In the present study, molecular characterization of fourteen *B. ovis* field isolates revealed a high genetic diversity among strains (Figure 1). Interestingly, among ten *B. ovis* isolates from same herd only two strains had identical patterns (Figure 1). The existence of many different genetic profiles within the same herd has two possible explanations: first, the existence of an intense animal traffic led the introduction of the agent from different origins and second, all *B. ovis* strains isolated from outbreak were originated from the same *B. ovis* strain that undergone some changes in *loci* of MLVA16. Although there are no epidemiological data that can confirm or refute the first explanation, the second hypothesis seems less likely, since the differences observed among the ten *B. ovis* strains from same herd were not the result of one-repeat unit increase or decrease and were also not restricted to only one MLVA16 *locus* or panel. Moreover, even though some data had suggested short term evolution particularly among panel 2B *loci*[27,28], there was also polymorphism at *locus* Bruce08 from the most conserved panel (panel 1) (Figure 1). On the other hand, in contrast to smooth strains such as *B. abortus, B. melitensis* and *B. suis* that have demonstrated a high stability of all MLVA16 *loci* under *in vivo* and *in vitro* conditions [12-14,29], MLVA16 performed on *B. canis,* a rough strain, suggesting a hypervariability particularly in some panel 2B *loci*[30]. Whole genome sequencing of these *B. ovis* strains from the same herd would be the better way to understand the biological significance of the high genetic diversity observed without any concerns, however it is less practical and much more expensive.

Clustering analysis also showed a large distance between the two isolates from Uruguaiana (Bruce09, 04, 07 and 16), and between the two *B. ovis* strains from undefined municipalities (Bruce08, 09, 07 and 16), likewise in comparison among all four isolates (Figure 1). These major differences in the MLVA16 genotypic profile and the large difference in the years of isolation of the strains (1982, 1985 and 1995) (Figure 2), together, strongly suggest that no epidemiological relationship exist among these four *B. ovis* isolates.

Minimal spanning tree analysis revealed the existence of two clearly distinct major clonal complexes (clonal complexes A and B) (Figure 2), one composed by most of Brazilian *B. ovis* isolates plus French strains and a single strain from Argentina, Australia, Spain and USA (clonal complex A), and a second one with fewer representatives and composed by two strains from France and a single strain from Argentina and Brazil (clonal complex B) (Figure 2). The establishment of these relationships is central to develop a model for evolutionary steps in the difference of the *B. ovis* MLVA16 genotypes. Nevertheless, more representative sampling is needed for inclusion into this model for a more robust comparison. Therefore, data of present study are especially important, because it expands the universe of *B. ovis* strains genotyped by MLVA16 in both, amount and origin of strains.

Moreover, since Rio Grande do Sul State is bordered by Argentina, the close relationship between *B. ovis* strain BCCN 98–46 from Argentina and the Brazilian *B. ovis* isolate 241E suggests that *B. ovis* strains were circulating in the Brazilian – Argentinean border. In this context, animal importation could also explain the very close localization of *B. ovis* isolates from Brazil and *B. ovis* strains from France and Spain in MST analysis. Although there are no recent records about importation of animals from these countries to Rio Grande do Sul, historical records show that the formation of the sheep flock of this State was mainly achieved through the importation of animals from various countries of Europe and Oceania [31,32]. Furthermore, the main activity of the flock from Santana do Livramento, RS, from where most *B. ovis* strains were isolated, was the rearing of Texel breeders, a breed whose origin is in France and the Germany.

In conclusion, the results of the present study showed a high genetic diversity among *B. ovis* field isolates from Rio Grande do Sul State, Brazil by MLVA16.

### Availability of supporting data

The data set supporting the results of this article is available in the Brucella_Brazil at http://mlva.u-psud.fr/brucella/ repository.

## Competing interests

The authors declare that they have no competing interests.

## Authors’ contributions

EMSD and GNF participated in design of the study, data acquisition and analysis. EMSD and wrote the paper. MGD, FPP and APL conceived and participated in design of the study, and critically reviewed the manuscript. All authors read and approved the final manuscript.
